# Detection of *Salmonella* spp. Using a Generic and Differential FRET-PCR

**DOI:** 10.1371/journal.pone.0076053

**Published:** 2013-10-16

**Authors:** Jilei Zhang, Lanjing Wei, Patrick Kelly, Mark Freeman, Kirsten Jaegerson, Jiansen Gong, Bu Xu, Zhiming Pan, Chuanling Xu, Chengming Wang

**Affiliations:** 1 Yangzhou University College of Veterinary Medicine, Yangzhou, Jiangsu, P. R. China; 2 Jiangsu Co-Innovation Center for the Prevention and Control of Important Animal Infectious Diseases and Zoonoses, Yangzhou, Jiangsu, P. R. China; 3 Ross University School of Veterinary Medicine, Basseterre, St. Kitts and Nevis; 4 Virginia-Maryland Regional College of Veterinary Medicine, Blacksburg, Virginia, United States of America; 5 Poultry Institute, Chinese Academy of Agricultural Sciences, Yangzhou, Jiangsu, P. R. China; 6 Yangzhou University College of Bioscience and Biotechnology, Yangzhou, Jiangsu, P. R. China; Cornell University, United States of America

## Abstract

To facilitate the detection of *Salmonella* and to be able to rapidly and conveniently determine the species/subspecies present, we developed and tested a generic and differential FRET-PCR targeting their tetrathionate reductase response regulator gene. The differential pan-*Salmonella* FRET-PCR we developed successfully detected seven plasmids that contained partial sequences of *S. bongori* and the six *S. enterica* subspecies. The detection limit varied from ∼5 copies of target gene/per PCR reaction for *S. enterica enterica* to ∼200 for *S. bongori*. Melting curve analysis demonstrated a *T*_m_ of ∼68°C for *S. enterica enterica*, ∼62.5°C for *S. enterica houtenae* and *S. enterica diarizonae*, ∼57°C for *S. enterica indica*, and ∼54°C for *S. bongori*, *S. enterica salamae* and *S. enterica arizonae*. The differential pan-*Salmonella* FRET-PCR also detected and determined the subspecies of 4 reference strains and 47 *Salmonella* isolated from clinically ill birds or pigs. Finally, we found it could directly detect and differentiate *Salmonella* in feline (5/50 positive; 10%; one *S. enterica salamae* and 4 *S. enterica enterica*) and canine feces (15/114 positive; 13.2%; all *S. enterica enterica*). The differential pan-*Salmonella* FRET-PCR failed to react with 96 non-*Salmonella* bacterial strains. Our experiments show the differential pan-*Salmonella* FRET-PCR we developed is a rapid, sensitive and specific method to detect and differentiate *Salmonella*.

## Introduction

*Salmonella* are important zoonotic pathogens which affect the health of people and animals worldwide. Advances in methods to detect *Salmonella* and a better understanding of their genomics have resulted in an improved classification system for the organisms. Currently, the genus *Salmonella* consists of only 2 species, *S*. *bongori* and *S*. *enterica*, with the latter containing 6 subspecies: *enterica* (I), *salamae* (II), *arizonae* (IIIa), *diarizonae* (IIIb), *houtenae* (IV), and *indica* (VI) [1], [2]. Using the White-Kauffmann-Le Minor Scheme based on somatic, flagellar and capsular antigens, around 2600 serovars are recognized of which over 99.5% belong to the *S. enterica* species and more than half are *S. enterica* subspecies *enterica* [1]. The *S. enterica enterica* serotypes are primarily associated with warm-blooded animals while *S. bongori* and the remaining five *S. enterica* subspecies are mainly found in cold-blooded animals and the environment [3]. While most human and animal infections are associated with *S. enterica enterica*, the growing popularity of reptiles as pets has led to an increasing number of infections with *S. bongori* and *S. enterica* subspecies other than *S. enterica enterica* [2], [4], [5], [6]. A retrospective analysis of over 75,000 isolates collected between 1985–2009 showed that *S. enterica* subspecies *salamae*, *arizonae*, *diarizonae* and *houtenae* caused invasive disease in people significantly more frequently than did *S. enterica enterica* [2].

The diagnosis of salmonellosis using conventional culture and serotyping is time consuming, laborious and requires large numbers of reagents. Effective nucleic acid-based methods could greatly simplify the procedure and a number of DNA-based techniques have been described to target specific *Salmonella* serovars [7]–[12]. Most commonly, they specifically identify *S. enterica* or only *S. enterica* subsp. *enterica* [13]–[19]. Although a multiplex PCR assay has been described to differentiate species and subspecies of *Salmonella* [20], the procedure requires organisms isolated in culture, is time consuming, and may be associated with decreased sensitivity and specificity due to complex interactions among five sets of primers. The FRET-PCR assay largely eliminates these problems and we therefore attempted to use this technique to design and test a differential pan-*Salmonella* FRET-PCR to sensitively and specifically detect and differentiate *Salmonella* species and subspecies. The ability to respire tetrathionate is a characteristic of *Salmonella* and tetrathionate broth is used as a standard enrichment medium for *Salmonella* species. The tetrathionate reductase locus (*ttr*), including the tetrathionate reductase response regulator gene (*ttrR*), is located within the *Salmonella* pathogenicity island 2 and is highly conserved with minimal genetic polymorphism among *Salmonella* species/subspecies. The inclusivity and exclusivity of the PCR technology targeting *ttrR* gene was found to be 100% while applying on 110 *Salmonella* strains and 87 non-*Salmonella* strains [16], [18]. This makes the *ttrR* an ideal target for molecular probes. The results of our experiments using the *ttrR* to develop a FRET-PCR to differentiate *Salmonella* are described in this report.

## Materials and Methods

### Fecal samples

The study was reviewed and approved by the Institutional Animal Care and Use Committee of the Ross University School of Veterinary Medicine (RUSVM). Between June and November of 2011, convenience free-catch fecal samples were obtained from dogs (n = 114) owned by consenting RUSVM veterinary students, and from stray cats (n = 50) involved in the RUSVM Small Animal Spay/Neuter Outreach Program. The animals from which feces were collected were clinically healthy and had no signs of diarrhea. Within 2 hours of collection into clean plastic fecal pots, around 1 g of the feces was transferred with a sterile spatula into a 2.0 ml sterile Eppendorf tube and frozen at −80°C until thawed at room temperature for DNA extraction.

### *Salmonella* strains

To test the sensitivity and specificity of the differential pan-*Salmonella* FRET-PCR, we used 47 *Salmonella* strains (Table 1) comprising 20 serotypes isolated from clinically ill chickens, ducks, geese, pigeons and pigs in five provinces of China between 1999–2013 [21], 4 reference strains [S. Typhimurium (ATCC 14028); *S.* Enteritidis (Gaertner) Castellani and Chalmers (ATCC 13076); *S.* Choleraesuis from Denmark (CMCC 50018); S. Gallinarum from China (CVCC 536)], and 96 non-*Salmonella* bacterial strains (84 *Escherichia coli* strains, 10 *Campylobacter* spp. strains, 1 *Pseudomonas aeruginosa* strain, and 1 *Enterococcus faecalis* strain). The Chinese samples from clinically ill birds and pigs had been grown on MacConkey agar, identified by biochemical characteristics (Biolog Inc., Hayward, California, USA) and serotyped using standard agglutination tests with O and H antisera (Tianrun, Ningbo, China).

**Table 1:** *Salmonella* strains used for selectivity of pan-*Salmonella* FRET-PCR.

Serotype	Time, host and location of *Salmonella* collection	*T*_m_
*S.* Indiana	2012; Goose; Danyang, Jiangsu, China	67.5°C
	2010; Goose; Yangzhou, Jiangsu, China	
	2011, Chicken; Yangzhou, Jiangsu, China	
	2012; Chicken; Xinxiang, Henan, China	
*S.* Potsdam	2010; Goose; Yangzhou, Jiangsu, China	68.0°C
	2012; Goose; Yancheng, Jiangsu, China	
	2012; Chicken; Yangzhou, Jiangsu, China	
	2009; Chicken; Yangzhou, Jiangsu, China	
*S.* Kottbus	2011; Duck; Tianchang, Anhui, China	68.0°C
*S.* Saintpaul	2010; Duck; Yangzhou, Jiangsu, China	68.0°C
	2009; Chicken; Yangzhou, Jiangsu, China	
*S.* Typhimurium	2012; Pigeon; Xinxiang, Henan, China	68.0°C
	2012; Goose; Yangzhou, Jiangsu, China	
	2008; Chicken; Taian, Shandong, China	
	2009; Chicken; Yangzhou, Jiangsu, China	
	2012; Pig; Nanjing, Jiangsu, China	
	ATCC 14028	
*S.* Heidelberg	2012; Goose; Danyang, Jiangsu, China	68.0°C
	2009; Chicken; Yangzhou, Jiangsu, China	
*S.* Reading	2009; Chicken; Yangzhou, Jiangsu, China	68.0°C
*S.* Derby	2009; Chicken; Yangzhou, Jiangsu, China	67.5°C
	2012; Pig; Nanjing, Jiangsu, China	
*S.* Enteritidis	2009; Chicken; Haian, Jiangsu, China	68.0°C
	1999; Duck; Xinjin, Sichuan, China	
	2003; Duck; Mianyang, Sichuan, China	
	2007; Duck; Weifang, Shandong, China	
	ATCC 13076	
*S.* Gullinarum	1956; Chicken, CVCC536 strain; Xian, Shanxi, China	68.0°C
*S.* Montevideo	2009; Chicken; Yangzhou, Jiangsu, China	68.5°C
*S.* Thompson	2009; Chicken; Yangzhou, Jiangsu, China	67.5°C
*S.* Bazenheid	2009; Chicken; Yangzhou, Jiangsu, China	68.0°C
*S.* Choleraesuis	CMCC 50018; Pig; Denmark	68.0°C
*S.* Kentucky	2009; Chicken; Yangzhou, Jiangsu, China	68.0°C
*S.* Blockley	2009; Chicken; Yangzhou, Jiangsu, China	68.5°C
*S.* Agona	2009; Chicken; Yangzhou, Jiangsu, China	66.5°C
*S.* Pullorum	2011–13; 10 strains from Chicken; Yangzhou, Jiangsu	68.5°C
	2009; 2 strains from Chicken; Tianchang, Anhui, China	
*S*. Anatis	2012; Pig; Nanjing, Jiangsu, China	66.0°C
*S*. Newport	2012; Pig; Nanjing, Jiangsu, China	68.0°C
*S*. Meleagridis	2012; Pig; Nanjing, Jiangsu, China	66.5°C

### Artificially inoculated dog feces

Two hundred µl of the ATCC strain *S.* Typhimurium 14028 with a concentration of 10^6^ CFU/ml was thoroughly mixed for 1 minute with 1 gram of *Salmonella* culture-negative canine feces suspended in 0.8 ml of sterile PBS. An aliquot of 100 µl was streaked onto MacConkey agar (Remel, Lenexa, Kansas, 66215, USA) and cultured for 18-hour culture at 37°C. As described below, the DNA was extracted from a further aliquot of 200 µl and tested with the differential pan-*Salmonella* FRET-PCR.

### Extraction of DNA from cat and dog feces

The QIAamp DNA Stool Mini Kit (Qiagen Inc., Valencia, CA, USA) was used to extract fecal DNA from cats and dogs following the manufacturer's instructions. Briefly, the fecal sample (100 mg) was lysed for 2 h at 55°C with buffer ATL and proteinase K before the reaction was stopped with buffer AL at 70°C for 10 min. After centrifugation, anhydrous ethanol was added to the supernatant and the mixture passed through the QIAamp kit column. Following two washes with buffers AW1 and AW2, DNA was eluted in 50 µL of elution buffer and stored at −80°C until thawed at room temperature for testing.

### Differential pan-*Salmonella* FRET-PCR

#### Primers and probes

**Figure 1:**
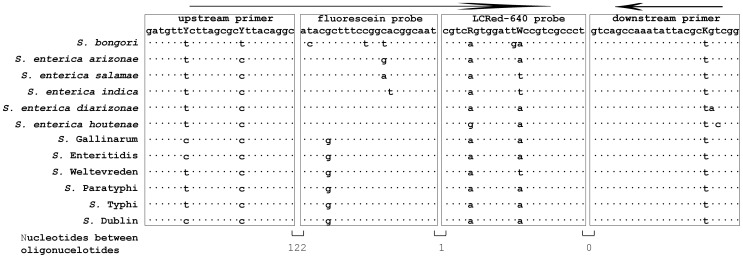
Alignment of primers and probes used in the differential pan-*Salmonella* FRET-PCR. Nucleotide sequences of the primers and probes are shown at the top of the figure and the *Salmonella* tested are shown down the side. Dots indicate that nucleotides are identical. The upstream primer and probes were used as shown while the downstream primer was used as a downstream oligonucleotide. The upstream and downstream primers and the LCRed 640 probe had 2, 1, and 2 degenerate nucleotides (shown in capital letters). The nucleotides between the primers and probes or between the probes are not shown.

**Figure 2:**
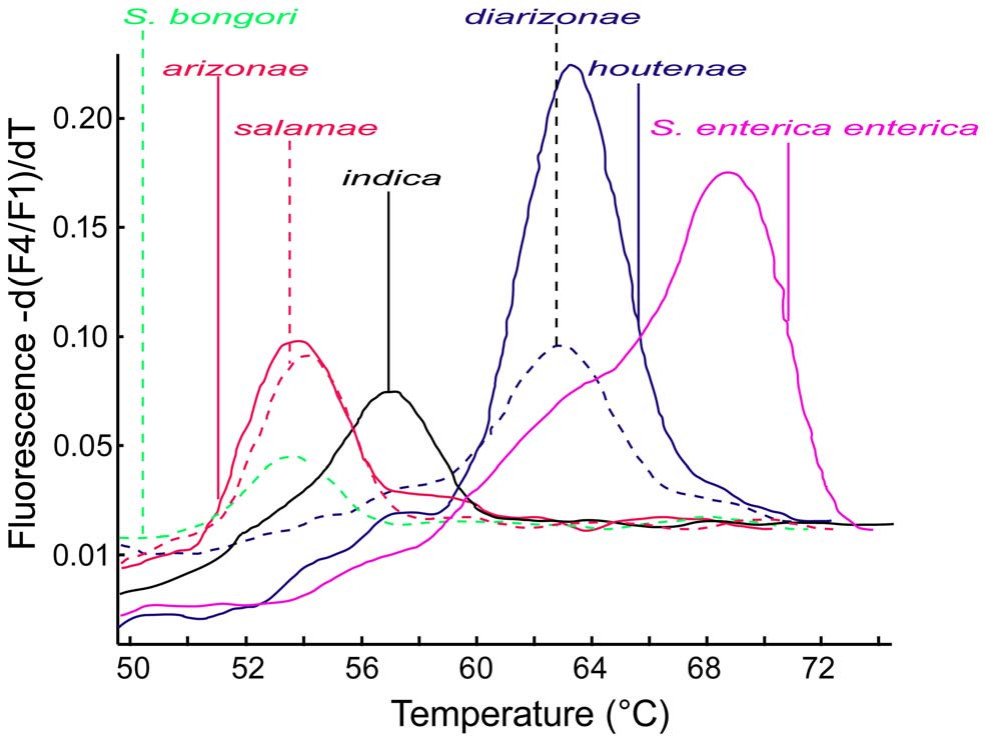
Melting curves of the pan-*Salmonella* FRET-PCR. Seven plasmids containing portions of *ttrR* gene of *S. bongori* and 6 *S. enterica* subspecies were used as the positive controls and for melting curve analysis of pan-*Salmonella* FRET-PCR. The difference in the numbers and types of nucleotide mismatches in the fluorescein and LCRed-640 probes we designed enabled us to identify 4 distinct groups of *Salmonella* based on their *T*_m_: *S. enterica* subsp. *enterica* has a *T*_m_ of ∼68°C (pink and solid line); ∼62.5°C for *S. enterica* subsp. *houtenae* (blue and solid line) and *S. enterica* subsp. *diarizonae* (blue and dashed line); ∼57°C for *S. enterica* subsp. *indica* (black and solid line); ∼54°C for *S. bongori* (green and dashed line), *S. enterica* subsp. *arizonae* (red and solid line), and *S. enterica* subsp. *salamae* (red and dashed line).

The *ttrR* sequences of *S. bongori*, six *S. enterica enterica* serovars (*S*. Gallinarium AM933173, *S*. Enteritidis AM933172, *S*. Weltevreden FR775221, *S*. Paratyphi CP000857, *S*. Typhi LT2 AF282268, *S*. Dublin CP001144) and five other *S. enterica* subspecies (*salamae*, *arizonae*, *diarizonae*, *houtenae*, *indica*) (Table 2) were obtained from GenBank (www.ncbi.nlm.nih.gov). The Clustal Multiple Alignment Algorithm was used to identify a highly conserved region of the *ttrR* gene common to all the above *Salmonella* (Figure 1). It was also used to identify probe regions that were relatively conserved but had a low level of polymorphism that would enable us to differentiate species and subspecies of *Salmonella* by high-resolution melting curve analysis (Figure 1, Figure 2). The primers and probes were synthesized by Integrated DNA Technologies (Coralville, IA, USA). The differential pan-*Salmonella* FRET-PCR we developed amplifies a 216-bp target and the positions of primers and probes are shown in Figure 1: forward primer: 5′-GATGTTYCTTAGCGCYTTACAGGC-3′; downstream primer: 5′-CCGACMGCGTAATATTTGGCTGAC-3′; anchor probe: 5′-ATACGCTTTCCGGCACGGCAAT-6-FAM-3′; reporter probe: 5′-LCRED640-CGTCRGTGGATTWCCGTCGCCCT-Phosphate-3′. The fluorescein probe was 3′-labeled with carboxyfluorescein (6-FAM) which acts as the FRET donor probe, excited by 488 nm light. The LCRed-640 probe was HPLC-purified and used as the FRET acceptor probe, emitting ∼640 nm fluorescence following excitation by 6-FAM in close physical proximity.

**Table 2:** Plasmids containing partial *Salmonella ttrR* gene used in this study.

*Salmonella* sequence contained in the plasmid	Gene accession number	Detection limit (copies/10 µL PCR reaction)	Melting temperature (*T*_m_)
*S. bongori*	AY578066	∼100	53°C
*S. enterica diarizonae*	AY578069	∼50	62°C
*S. enterica houtenae*	AY578068	∼50	62.5°C
*S. enterica arizonae*	CP000880	∼100	53.5°C
*S. enterica indica*	AY578065	∼100	57°C
*S. enterica salamae*	AY578070	∼100	54°C
*S. enterica enterica* Typhimurium	AF282268	∼5	68°C

#### Plasmids

Seven plasmids (Integrated DNA Technologies, Coralville, IA, USA) containing portions of the ttrR gene of S. bongori, S. enterica enterica, S. enterica salamae, S. enterica arizonae, S. enterica diarizonae, S. enterica houtenae and S. enterica indica were used as the positive controls and for quantitative standards (Table 2). Seven nucleotide fragments representing partial ttrR regions of the above Salmonella were synthesized and inserted in the pIDTSMART cloning Vector (Integrated DNA Technologies, Coralville, IA, USA). The resulting plasmids were linearized with HindIII (Promega, Madison, WI, USA) and quantified by PicoGreen® DNA fluorescence assay (Molecular Probes, Eugene, OR, USA) for preparation of quantitative standards (10^4^, 10^3^, 10^2^, 10^1^, 10^0^ copies of ttrR molecules/10 µL).

#### Thermal cycling

Differential pan-*Salmonella* FRET-PCR was performed in a LightCycler® 2.0 real-time PCR platform using a thermal protocol and PCR conditions as described [22]. Each reaction was performed in a 20 µL final volume containing 10 µL of extracted DNA. Thermal cycling consisted of 18 high-stringency step-down cycles followed by 30 relaxed-stringency fluorescence acquisition cycles. The 18 high-stringency step-down thermal cycles were 6×0 sec @ 95°C, 12 sec @ 64°C, 8 sec @ 72°C; 9×0 sec @ 95°C, 12 sec @ 62°C, 8 sec @ 72°C; 3×0 sec @ 95°C, 12 sec @ 60°C, 8 sec @ 72°C. The relaxed-stringency fluorescence acquisition cycling consisted of 30×0 sec @ 95°C, followed by fluorescence acquisition of 12 sec @ 56°C, and 10 sec @ 72°C. Following the completion of differential pan-*Salmonella* FRET-PCR, the melting curve analysis for probes annealing to the PCR products was determined by monitoring the fluorescence from 45°C to 80°C, and the first derivatives of F4/F1 were evaluated to determine the probe melting temperature (*T*_m_). This setting is only possible on LightCycler 1.5 and 2.0 models which can accommodate very rapid temperature ramps because of their capillary tube reaction system.

PCR products were verified using electrophoresis through 4% MetaPhor agarose gels and purified for automated DNA sequencing with a QIAquick PCR Purification Kit according to the manufacturer's instructions (Qiagen, Valencia, CA, USA). Both strands of DNA of the PCR products were sequenced at the Genomic Sequencing Laboratory (Davis Sequencing, Davis, USA) using the forward and downstream primers (Figure 1).

## Results

### Development of the differential pan-*Salmonella* FRET-PCR

Our differential pan-*Salmonella* FRET-PCR detected each of the 7 plasmids containing partial *ttrR* sequences of *S. bongori*, the 6 *S. enterica* subspecies (Table 2). It also detected all 47 *Salmonella* isolates made from clinically ill birds and pigs in China and the four reference strains; the *T*_m_ for these isolates varied from 66.5°C to 68.5°C (Table 1). No reaction products were obtained when the differential pan-*Salmonella* FRET-PCR was performed on 96 strains of non-*Salmonella* bacteria.

Using the *Salmonella ttrR*-containing plasmids as quantitative standards, we determined that the detection limit of the FRET-PCR varied from ∼5 copies of the *S. enterica enterica ttrR* gene per PCR reaction to ∼200 copies per PCR reaction for *S. bongori* (Table 2). Melting curve analysis enabled a high level of differentiation of *Salmonella* with strains clustering in four groups based on their melting points: i) *S. enterica* subsp. *enterica* had a *T*_m_ of ∼68°C; ii) ∼62.5°C for *S. enterica* subsp. *houtenae* and *S. enterica* subsp. *diarizonae*; iii) ∼57°C for *S. enterica* subsp. *indica*; iv) ∼54°C for *S. bongori*, *S. enterica* subsp. *salamae*, and *S. enterica* subsp. *arizonae* (Figure 2). High-resolution melting analysis on samples containing mixtures of plasmids revealed multiple distinct melting peaks that enabled us to differentiate the *Salmonella* species/subspecies involved (data not shown).

### Artificially inoculated dog feces

Following incubation of the fecal sample typical black *Salmonella* colonies were observed on the inoculated MacConkey agar and confirmed to be S. Typhimurium by standard biochemical testing. Differential pan-*Salmonella* FRET-PCR of the fecal sample was positive with a *T*_m_ of 68°C which was consistent with *S*. Typhimurium.

### Direct detection of fecal *Salmonella* DNA by the differential pan-*Salmonella* FRET-PCR

Of the 164 fecal samples studied, 10% of the feline specimens (5/50) and 13.2% of the canine specimens (15/114) were positive for *Salmonella* by the differential pan-*Salmonella* FRET-PCR. Of the positive specimens, 95% (19/20) were *S. enterica enterica* based on their *T_m_* of ∼68°C and this was confirmed by gel electrophoresis and sequencing (Figure-S1; Figure-S2). One fecal DNA from a cat had a *T_m_* of 54°C which was consistent with *S. enterica salamae* and this was also confirmed by sequencing of the reaction products.

## Discussion

By systematically aligning the *ttrR* sequences of representative *Salmonella* species, subspecies and serovars, we were able to identify a region suitable for targeting in a differential pan-*Salmonella* FRET-PCR. The primers we designed proved to be highly conserved and enabled us to amplify all the *Salmonella* species and subspecies we tested, including four reference strains and 47 isolates made from clinically ill birds and pigs. We were unable, however, to amplify non-*Salmonella* bacteria which shows our test has a high specificity. The probes we designed were also conserved but had a low level of polymorphism with the different *Salmonella* species and subspecies which resulted in different melting points for the organisms. Based on their melting points we were able to rapidly (under 4 hours from DNA extraction to melting curve analysis) and clearly detect and differentiate *S. enterica enterica* which is the most important subspecies, and in addition the less common and important pathogen *S. enterica indica*. Although our test did not enable us to differentiate between *S. enterica* subsp. *houtenae* and *S. enterica* subsp. *diarizonae*, and also between *S. bongori*, *S. enterica* subsp. *salamae*, and *S. enterica* subsp. *arizonae*, these organisms are only isolated comparatively rarely in clinical samples [3]. In some areas, however, they are being recognized more frequently [2] and once the possible subspecies have been found in our FRET-PCR, differentiation between them would require sequencing of the reaction products. If biochemical tests are to be used to make the identification, knowledge of the potential subspecies gained from the FRET-PCR would enable more specific testing to be performed.

While a multiplex PCR has been described which identifies all the *Salmonella* species and subspecies, the test requires cultures of organisms and uses 5 primers [20], the products of which need to be resolved on gels by electrophoresis. Our differential pan-*Salmonella* FRET-PCR requires only a single set of primers and one set of probes, and the products are revealed as the differential pan-*Salmonella* FRET-PCR assay is performed. This greatly simplifies the procedure and assures the sensitivity and specificity of *Salmonella* detection, and the rapidity of the test means it will be very useful in situations where screening of large numbers of samples is required in a timely and convenient fashion. Compared to the classical gel-based PCR, our differential pan-*Salmonella* FRET-PCR might be more expensive but has the advantages of reducing labor costs, eliminating the need for electrophoresis equipment, gels and expensive biohazard disposal, and, most importantly, in minimizing the chances of carry-over contamination.

Numerous PCR assays have been described for the detection of *Salmonella* DNA in a variety of samples, food in particular [7]–[10], [13]–[20]. As with routine cultures, however, these PCR methods described to date for *Salmonella* detection usually require a pre-enrichment step which delays testing by 6 to 24 hours [19], [23], and make the tests unsuitable for rapid screening of large numbers of samples. Our differential pan-*Salmonella* FRET-PCR was effective in directly detecting *Salmonella* DNA in experimentally inoculated dog feces when we tested a sample containing approximately 10^5^ organisms, at least some of which were viable and could be cultured. This experiment indicates the differential pan-*Salmonella* FRET-PCR is sensitive and capable of differentiating its target in a complex matrix containing a large variety of microbial flora and host genome. We were also able to use the differential pan-Salmonella FRET-PCR to detect *Salmonella* in dog and cat feces from apparently healthy animals. Although we did not culture the feces from the dogs and cats and were therefore unable to precisely determine the sensitivity and specificity of the test when performed directly on fecal samples, our findings are comparable with other studies using PCR which found *Salmonella* in 6% and 2% of dogs [24] and cats [25] with normal feces, respectively. Further studies are underway in our laboratory to determine the sensitivity of the PCR in detecting DNA of *Salmonella* directly in tissue, fecal and environmental samples.

In conclusion, our study established a novel differential pan-*Salmonella* FRET-PCR with high sensitivity and specificity to quantitatively detect all *Salmonella* species and subspecies. Melting curve analysis following amplification and real-time detection conclusively and conveniently differentiated *Salmonella* species and subspecies and enabled us to diagnose, for the first time, the presence of *S. enterica salamae* in the feces of a clinically healthy cat.

## Supporting Information

Figure S1 **Representative melting curves of the pan-** ***Salmonella*** **FRET-PCR of canine fecal samples.** DNA extracted from canine fecal samples was used for the pan-*Salmonella* FRET-PCR described in this study. The positive samples showed an identical *T*_m_ of 68°C while the melting curves of negative samples remained flat.
(TIF)

Figure S2 **Gel electrophoresis (4.0% agarose) analysis of the pan-** ***Salmonella*** **FRET-PCR's amplified products.** Lane 1: Trans2K Plus DNA Marker (Beijing Transgen Biotech Co., Ltd.); Lanes 2–3: positive fecal samples from cats; Lane 4: positive fecal sample from dog; Lanes 5–11: 7 plasmids containing part of *ttrR* gene in the following order: *S. enterica enterica*, *S. enterica houtenae*, *S. enterica diarizonae*, *S. enterica indica*, *S. enterica salamae*, *S. enterica Arizonae* and *S. bongori*; Lanes 12–13: negative fecal samples from cat and dog; Lanes 14–17: DNAs extracted from *Escherichia coli*, *Campylobacter* spp., *Pseudomonas aeruginosa*, and *Enterococcus faecalis*, respectively.
(TIF)
