# Supplementary figures and images for: Detection of Salmonella spp. Using a Generic and Differential FRET-PCR
Source: PLoS One. 2013 Oct 16;8(10):e76053. doi: 10.1371/journal.pone.0076053 (PMC3797804; doi:10.1371/journal.pone.0076053)

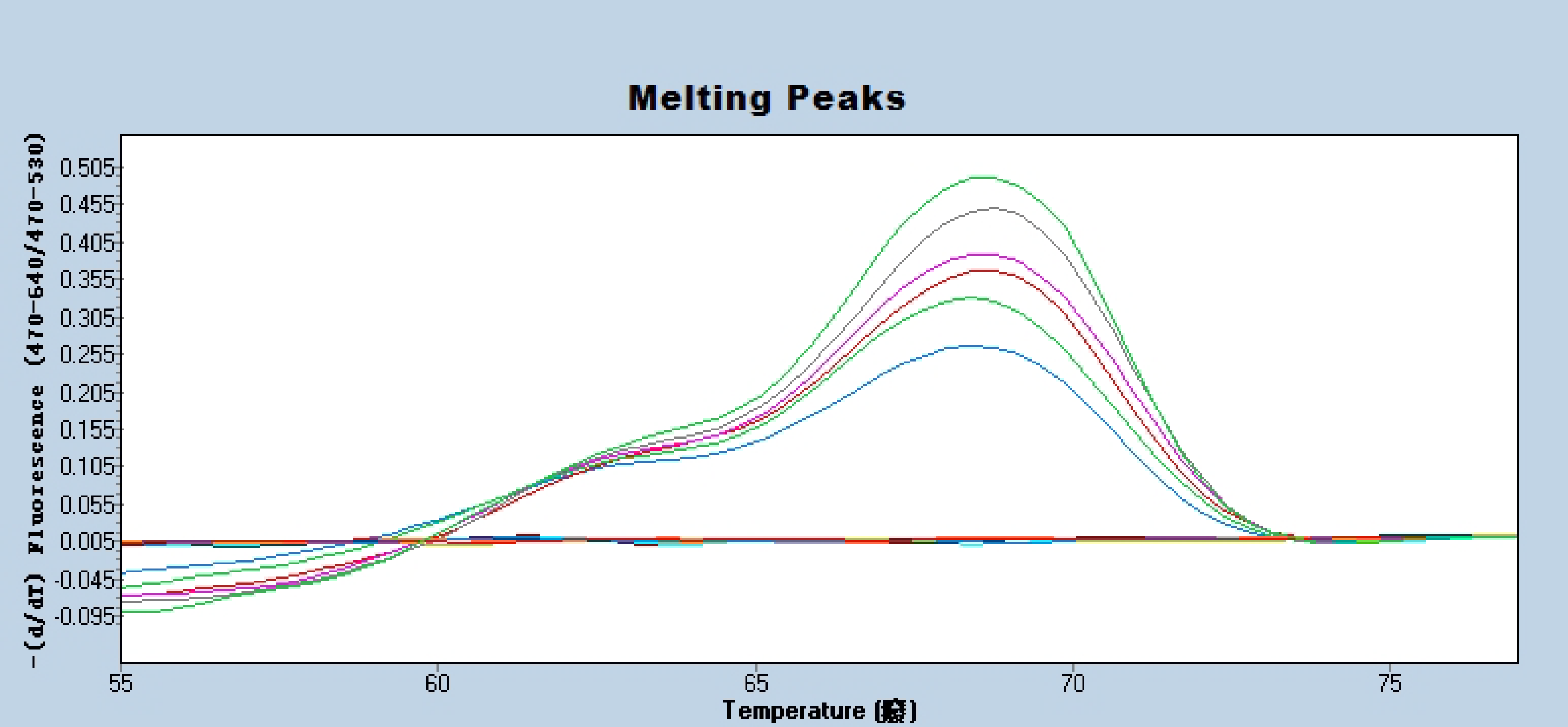

Supplement: Figure S1 — Representative melting curves of the pan- Salmonella FRET-PCR of canine fecal samples. DNA extracted from canine fecal samples was used for the pan-Salmonella FRET-PCR described in this study. The positive samples showed an identical T m of 68°C while the melting curves of negative samples remained flat. (TIF) [file pone.0076053.s001.tif]

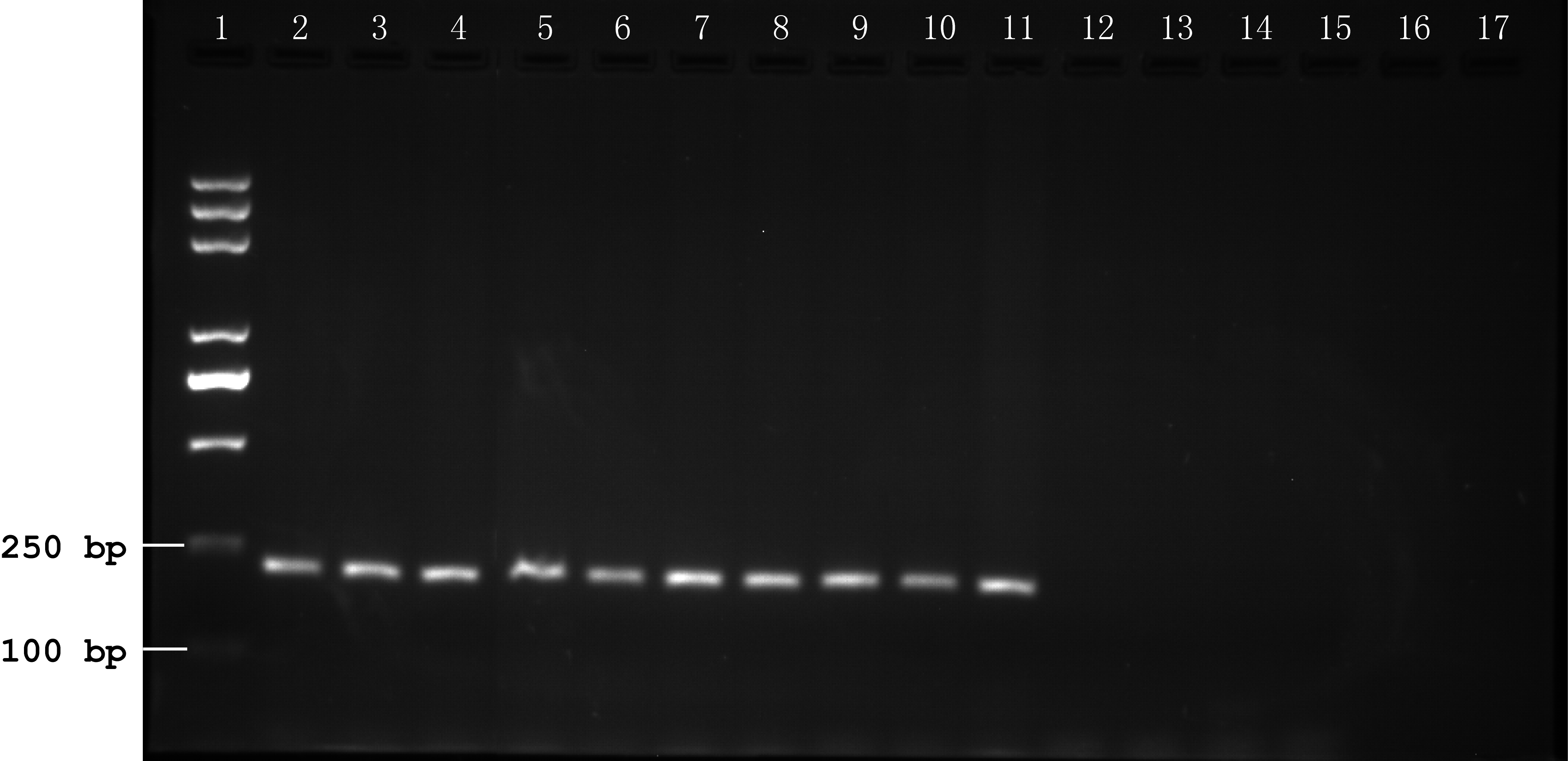

Supplement: Figure S2 — Gel electrophoresis (4.0% agarose) analysis of the pan- Salmonella FRET-PCR's amplified products. Lane 1: Trans2K Plus DNA Marker (Beijing Transgen Biotech Co., Ltd.); Lanes 2–3: positive fecal samples from cats; Lane 4: positive fecal sample from dog; Lanes 5–11: 7 plasmids containing part of ttrR gene in the following order: S. enterica enterica, S. enterica houtenae, S. enterica diarizonae, S. enterica indica, S. enterica salamae, S. enterica Arizonae and S. bongori; Lanes 12–13: negative fecal samples from cat and dog; Lanes 14–17: DNAs extracted from Escherichia coli, Campylobacter spp., Pseudomonas aeruginosa, and Enterococcus faecalis, respectively. (TIF) [file pone.0076053.s002.tif]
